# Psychological health is associated with knee pain and physical function in patients with knee osteoarthritis: an exploratory cross-sectional study

**DOI:** 10.1186/s40359-018-0234-3

**Published:** 2018-05-02

**Authors:** Hirotaka Iijima, Tomoki Aoyama, Naoto Fukutani, Takuya Isho, Yuko Yamamoto, Masakazu Hiraoka, Kazuyuki Miyanobu, Masashi Jinnouchi, Eishi Kaneda, Hiroshi Kuroki, Shuichi Matsuda

**Affiliations:** 1 Department of Physical Therapy, Human Health Sciences, Graduate School of Medicine, Kyoto University, Kyoto, Japan; 2 Japan Society for the Promotion of Science, Tokyo, Japan; 3 Department of System Design Engineering, Keio University, Yokohama, Japan; 4 Rehabilitation Center, Fujioka General Hospital, Gunma, Japan; 5 Nozomi Orthopaedic Clinic, Hiroshima, Japan; 6 Nozomi Orthopaedic Clinic Studium, Hiroshima, Japan; 7 Nozomi Orthopaedic Clinic Hiroshima, Hiroshima, Japan; 8 Department of Orthopaedic Surgery, Graduate School of Medicine, Kyoto University, Kyoto, Japan

**Keywords:** Knee osteoarthritis, Depression, Knee pain, Functional limitation, Physical activity

## Abstract

**Background:**

Depressive symptoms are a major comorbidity in older adults with knee osteoarthritis (OA). However, the type of activity-induced knee pain associated with depression has not been examined. Furthermore, there is conflicting evidence regarding the association between depression and performance-based physical function. This study aimed to examine (i) the association between depressive symptoms and knee pain intensity, particularly task-specific knee pain during daily living, and (ii) the association between depressive symptoms and performance-based physical function, while considering other potential risk factors, including bilateral knee pain and ambulatory physical activity.

**Methods:**

Patients in orthopaedic clinics (*n* = 95; age, 61–91 years; 67.4% female) who were diagnosed with radiographic knee OA (Kellgren/Lawrence [K/L] grade ≥ 1) underwent evaluation of psychological health using the Geriatric Depression Scale (GDS). Knee pain and physical function were assessed using the Japanese Knee Osteoarthritis Measure (JKOM), 10-m walk, timed up and go (TUG), and five-repetition chair stand tests.

**Results:**

Ordinal logistic regression analysis showed that depression, defined as a GDS score ≥ 5 points, was significantly associated with a worse score on the JKOM pain-subcategory and a higher level of task-specific knee pain intensity during daily living, after being adjusted for age, sex, body mass index (BMI), K/L grade, and ambulatory physical activity. Furthermore, depression was significantly associated with a slower gait velocity and a longer TUG time, after adjusting for age, sex, BMI, K/L grade, presence of bilateral knee pain, and ambulatory physical activity.

**Conclusions:**

These findings indicate that depression may be associated with increased knee pain intensity during daily living in a non-task-specific manner and is associated with functional limitation in patients with knee OA, even after controlling for covariates, including bilateral knee pain and ambulatory physical activity.

**Electronic supplementary material:**

The online version of this article (10.1186/s40359-018-0234-3) contains supplementary material, which is available to authorized users.

## Background

Osteoarthritis (OA) of the knee, a leading cause of pain and physical impairment [1], is the most common type of arthritis among older adults [2]. Recently, the importance of depressive symptoms in individuals with knee OA has gained increased recognition [3]. Depressive symptoms are a major comorbidity in older adults with knee OA with prevalence rate of 20% [4], which is higher than the prevalence in the general US population [5]. Depression symptoms have been suggested to be inversely associated with both knee pain and self-reported physical function [6, 7]. The importance of depressive symptoms in individuals with knee OA is further evidenced by the observation that treating depression in patients with knee OA reduces knee pain and improves self-reported physical function [8]. However, the type of activity-induced knee pain that is associated with depression has not been examined. Weight-bearing pain is suggested to represent a different aspect of knee pain compared to non-weight-bearing pain [9]. Since the impact of knee pain on individuals’ daily activities differs by the type of activity [10], a better understanding of the relationship between depression and task-specific knee pain would provide a comprehensive understanding of the depression-pain link that may help clarify the mechanism by which depressive symptoms cause exacerbation of knee pain, or vice versa in individuals with knee OA.

There is conflicting evidence regarding the association between depression and performance-based physical function. Some investigators have reported a significant association [11, 12], while others have found none [13–15]. This may be due to potential risk factors for performance-based physical function, such as bilateral knee pain [16, 17] and objectively measured physical activity [18, 19], not being assessed in previous studies. Patients with unilateral knee pain can compensate with the healthy knee to complete functional tasks. Thus, patients with bilateral knee pain are suggested to more likely be impaired in performance-based physical function [16, 17]. Although one study considered self-reported physical activity as a covariate on the association between depression and performance-based physical function [11], self-reported physical activity may overestimate physical activity compared to objectively measured physical activity [20].

Thus, the purpose of present exploratory cross-sectional study was to examine (i) the association between depressive symptoms and knee pain intensity, particularly task-specific knee pain during daily living, and (ii) the association between depressive symptoms and performance-based physical function, while considering potential risk factors, including bilateral knee pain and objectively measured ambulatory physical activity. We hypothesized that (i) individuals with depression had worse knee pain regardless of weight- and non-weight-bearing pain, and that (ii) the positive association between depression and worse performance-based functional measures was achieved through covariates, including bilateral knee pain and objectively measured ambulatory physical activity.

## Methods

### Participants

This was an exploratory cross-sectional study. The ethical committee of the affiliated institution approved the study (approval number: E1923). This cross-sectional study included outpatients with knee OA from community orthopaedic clinics in Hiroshima, Japan, who were identified through the medical record system. An advertisement was distributed to patients who sought conservative treatment for knee OA in January 2015.

The eligibility criteria included: (i) age ≥ 50 years; (ii) knees with radiographic OA (i.e., Kellgren/Lawrence [K/L] [21] grade ≥ 1) in one or both knees, as evaluated by weight-bearing anteroposterior radiographs; and (iii) an ability to walk independently on a flat surface without any ambulatory assistive device. The exclusion criteria were the following: (i) a history of knee surgery, (ii) inflammatory arthritis, (iii) periarticular fracture, or (iv) neurological problems. Since pre-radiographically defined knee OA, particularly of K/L grade 1, predicts radiographic OA progression to at least grade 2 [22, 23], we included patients with K/L grades ≥1. Patients with either bilateral or unilateral knee OA were considered.

### Measures

Clinical data, except radiographic data, were collected in one session. For all patients, the following outcome measurements were evaluated: Geriatric Depression Scale (GDS) score, a knee OA-related health domain measure (the Japanese Knee Osteoarthritis Measure [JKOM]), and three functional performance measurements (the 10 m walk, timed up and go [TUG], and five-repetition chair stand [5CS]). Demographic characteristics, radiographic OA severity, bilateral knee pain, and objectively measured ambulatory physical activity were assessed as covariates.

#### Evaluation of psychological health: GDS

Depressive symptoms were evaluated using the 15-item version of the GDS (range 0–15) [24], which is a standardized self-questionnaire (response: yes or no). Higher scores indicate more depressive symptoms (0 point indicates no depression and 15 points indicates severe depression). The GDS score is now one of the most widely used depression scales in the older population [25]. Mild depression was defined as score of ≥5 points, and moderate/severe depression was defined as score of ≥11 points [25–27].

#### Knee OA-related health domain measure: JKOM

The JKOM is a patient-based, self-answered evaluation scoring system that assesses “pain and stiffness” (8 questions, 0–32 points), “activities of daily living” (10 questions, 0–40 points), “participation in social activities” (5 questions, 0–20 points), and “general health conditions” (2 questions, 0–8 points), with a maximum score of 100 points in a person-specific assessment. In this study, only the JKOM “pain and stiffness” and “activities of daily living” scores were used. For each subscale, higher scores indicate a worse condition (response: 0–4 points; 0 indicates no pain or difficulty and 4 represents extreme pain or difficulty). The concurrent and construct validity of the JKOM was established by comparing with the WOMAC and the Medical Outcomes Study 36-item Short-Form Health Survey [28]. Cronbach’s alpha coefficient was 0.911 for the JKOM all items [28].

#### Performance-based physical function measures

We assessed objective performance-based physical function using identified activities recommended by the Osteoarthritis Research Society International (OARSI), as follows: gait velocity (short-distance walking), time of TUG (ambulatory transitions) and 5CS (sit-to-stand). Patients were instructed to walk 10 m at comfortable speed. We measured the time with a stop watch and the number of steps required to walk 10 m [29]. Subsequently, gait velocity (meters/second) was manually calculated. The TUG test [30], a simple, common, and reliable test for clinical use in individuals with or at risk of developing knee OA, was performed [31]. Patients were instructed to rise from a chair, walk 3 m, turn around, return, and sit down as fast as possible. The time was measured using a stopwatch. Furthermore, the 5CS test, which measures the time required for 5 repetitions of rising from a chair and sitting down as fast as possible, was evaluated. The TUG and 5CS tests can be feasibly used by clinicians [32].

#### Assessment of covariates

Data on age, sex, and height were self-reported by patients. Weight was measured on a scale, with the participants wearing their clothes without shoes. Body mass index (BMI) was calculated by dividing the weight by the square of height.

Radiographic OA severity of the “index knee” in each patient was assessed in the anteroposterior short view in the weight-bearing position using the K/L grading system [21]. The index knee was defined as the more painful knee in either the past or present. If patients felt that their knees were equally painful, the index knee was selected randomly using computer-generated permuted block randomization scheme [33]. The OA severity in the tibiofemoral joint was assessed by two trained examiners (HI and TA). To assess intra-rater and inter-rater reliability scores, 100 randomly selected radiographs were scored again by the same examiner more than 1 week after the first assessment. Both intra-rater and inter-rater reliability scores were excellent (intra-rater: κ = 0.88, 95% CI = 0.83, 0.92; inter-rater: κ = 0.84, 95% CI = 0.79, 0.90).

Bilateral knee pain was assessed using a questionnaire. Patients were asked: “In which knee do you have pain? Right? Left? Both?” Patients who answered “both” to this question were defined as having bilateral knee pain.

Objective ambulatory physical activity (steps/day) was assessed by measuring the daily, accumulated step counts using a pedometer (Yamax Power Walker EX-300; Yamasa Tokei Keiki Co., Ltd., Tokyo, Japan). This pedometer gives a mean step count within 3% of actual steps [34] and validated in free-living conditions [35]. We selected a pedometer, because it is cheap, readily accessible, and more likely to be used in clinical and public health applications. Each patient received a pedometer with instructions and an activity calendar for recording data. Patients were asked to wear the pedometer in the pocket of their dominant leg for 14 consecutive days, and removed it when bathing, sleeping, or performing water-based activities. The participants were asked to record the number of steps at the end of each day, and completed activity calendars were returned via mail after 14 consecutive days. The sample was restricted to patients who wore the pedometer for at least 10 days, which is more than time enough to reliably estimate physical activity (i.e., 3 days) [36]. We then calculated the average steps/day.

### Statistical analyses

Because this study is an exploratory study, rather than a hypothesis testing study, the sample size was not estimated before conducting the study (i.e., January 2015). The number of eligible patients attending the clinics during the study period was determined as the sample size.

Data analyses were performed with JMP Pro 12.2 (SAS Institute, Cary, NC, USA). To examine reliability of the JKOM, Cronbach’s alpha was calculated. As Cronbach’s alpha is a property of the scores on a test from a specific sample of participants [37], Cronbach’s alpha was estimated in this study’s participants. JKOM “pain and stiffness” and “activities of daily livings” were different domains detected by factor analysis [28]; therefore, Cronbach’s alpha for each domain was estimated.

Patients were categorized into two groups: depression (GDS score ≥ 5 points) or no depression (GDS score < 5). Each outcome variable was statistically compared between patients with and without depression. In these comparisons, univariate analyses were performed using Student’s *t*-test for parametric continuous variables, the Mann-Whitney *U* test for nonparametric continuous variables, and the chi-square/Fisher’s exact test for dichotomous/categorical variables. The normality of continuous variables was assessed with the Shapiro-Wilk test. The homogeneity of the variances between groups for all parametric continuous variables was confirmed using the Levene’s test. Descriptive statistics were calculated as means and standard deviations (SD) for continuous variables, and as proportions for dichotomous/categorical variables.

To evaluate the association between depression symptoms, knee pain intensity and functional measures, we performed an ordinal logistic regression analysis with knee pain intensity (JKOM “pain and stiffness” summated score) and each functional measure (JKOM “activities of daily living” summated score, gait velocity, TUG, and 5CS) as dependent variables and depression (0 = no depression, 1 = depression) as an independent variable. Ordinal logistic regression is a model for ordinal categorical outcome variables and works for skewed continuous outcome variables using ranks of data [38]. In the ordinal logistic regression models, each dependent variable was categorized into four groups by quartiles (Additional file 1: Table S1) and treated as ordinal variables (1–4; 1 [< 25th percentile] indicates mild pain or better function and 4 [≥75 percentile] indicates severe pain or worse function). Proportional odd ratio (OR) and 95% confidence intervals (CIs) for a greater quartile of each outcome measure was calculated to indicate predictive ability of depression while simultaneously including (one-step model) age (continuous), sex, body mass index (continuous), tibiofemoral joint K/L grade (continuous), and ambulatory physical activity (continuous) in the ordinal regression model. In the ordinal regression model in which the functional measures were included as dependent variables, bilateral knee pain (0: absence, 1: presence) was further included as a covariate.

Subsequently, further ordinal logistic regression analysis was performed to examine the association of depression with individual questions (i.e., 8 items) of the JKOM “pain and stiffness” sub-category. Since few of the individual pain scores were high, individual scores of 2, 3, and 4 were combined into one level (moderate/severe pain), and included in the ordinal logistic regression model as a dependent variable (0: no pain, 1: mild pain, 2: moderate/severe pain), as applied to WOMAC pain questions [39]. Assumption of proportional OR was also checked before all analyses. In these analyses, covariates were also included as mentioned above. These covariates were chosen a priori based on clinical judgment for possibly being associated with depression and knee pain or physical function and not on the causal pathway [16, 17, 40–42]. All independent variables were screened for collinearity by calculating bivariate Spearman correlation coefficients. Results of lack of fit (goodness of fit) test was checked to be non-significant if there is little to be gained by introducing additional variables such as polynomials and crossed terms. Overall model evaluation was done by checking the results of whole model test provided in JMP Pro 12.2. We checked the maximum number of independent variables included in the ordinal logistic regression model. The maximum number of independent variables included in the ordinal logistic regression model was determined based on the following formula:

1 $$ \left(n\frac{1}{n^2}\sum \limits_{i=1}^k{n}_i^3\right)/15 $$

k: number of categories, n: total sample size, n_i_: sample size in each category.

Since this is an exploratory study, the type I error rate was not adjusted for multiple comparisons of logistic regression analyses as endorsed by the European Agency for the Evaluation of Medicinal Products [43]. *p*-values < 0.05 were considered statistically significant.

## Results

We enrolled 102 patients initially; however, seven patients were excluded due to missing outcome variables. The remaining 95 patients (age, 61–91 years; 67.4% female) with K/L grade ≥ 1 (93.1% of the initial cohort) were included in the final analysis. Of 95 patients, 43 (45.3%) had depression (i.e., GSD score ≥ 5 points), of which 41 and 2 patients had mild and moderate/severe depression, respectively. Table 1 summarizes patients’ characteristics in patients with and without depression. Importantly, patients with depression had a significantly higher proportion of bilateral knee pain (*p* = 0.035), worse score of JKOM “pain and stiffness” (*p* = 0.004) and “activities of daily living” (*p* = 0.001), slower gait velocity (*p* = 0.017), and longer TUG time (*p* = 0.028). Cronbach’s alpha coefficients were 0.955 and 0.912 for JKOM “pain and stiffness” and “activities of daily living”, respectively.

**Table 1:** Demographic characteristics, osteoarthritis severity, objectively measured physical activity, knee pain, physical function, and psychological health in patients *with* and *without* depression (*n* = 95)

Variables	*With* Depression (*n* = 43)	*Without* depression (*n* = 52)	*p*-value*
	Mean ± SD or n (%)	Mean ± SD or n (%)	
Age, years	75.3 ± 7.31	74.3 ± 7.91	0.463
Female	31 (72.1)	33 (63.5)	0.390
Body mass index, kg/m^2^	24.0 ± 3.32	24.1 ± 3.75	0.952
Index knee tibiofemoral joint K/L grade			
Grade 1	15 (34.9)	17 (32.7)	0.343
Grade 2	15 (34.9)	23 (44.2)	
Grade 3	7 (16.3)	10 (19.2)	
Grade 4	6 (13.9)	2 (3.8)	
Ambulatory physical activity, steps/day	4950 ± 2390	4073 ± 2661	0.075
*Pain*			
Presence of bilateral knee pain	22 (51.2)	15 (28.8)	**0.035**
JKOM “pain and stiffness” (0–32 points)	9.91 ± 7.01	6.25 ± 7.11	**0.004**
*Self-reported physical function*			
JKOM “activities of daily living” (0–40 points)	9.93 ± 7.17	6.13 ± 7.41	**0.001**
*Performance-based physical function*			
Gait velocity, meters/second	1.03 ± 0.22	1.13 ± 0.20	**0.017**
Timed up and go, seconds	9.03 ± 2.97	7.91 ± 2.07	**0.028**
Five repetition chair stand, seconds	9.17 ± 1.86	8.58 ± 2.23	0.117
*Psychological health*			
Geriatric depression scale (0–15 points)	7.33 ± 1.87	1.8 ± 1.44	**–**
Normal (0–4 points)	–	52 (100.0)	
Mild depression (5–10 points)	41 (95.3)	–	
Moderate/severe depression (11–15 points)	2 (4.7)	–	

*K/L grade* Kellgren/Lawrence grade; *JKOM* Japanese Knee Osteoarthritis Measure

* Based on unadjusted analysis (Student *t*-test [gait velocity] or Mann-Whitney *U*-test [age, body mass index, ambulatory physical activity, JKOM score, timed up and go, and five repetition chair stand] or Fisher’s exact tests [female, index knee K/L grade, presence of bilateral knee pain]) between patients with and without depression. Non-normality of continuous variables, analysed using Mann-Whitney *U*-test, are assessed with the Shapiro-Wilk test (*p* < 0.05). Bold represents statistically significant result

Ordinal logistic regression analysis (Table 2) demonstrated that depression was significantly associated with a higher odds ratio of a greater quartile (i.e., severe pain) in the JKOM “pain and stiffness” (proportional OR: 3.01; 95% CI: 1.37, 6.62; *p* = 0.006) after being adjusted for age, sex, BMI, K/L grade, and ambulatory physical activity. Furthermore, depression was significantly associated with a higher odds ratio of a greater quartile in individual questions from the JKOM “pain and stiffness” after being adjusted for age, sex, BMI, K/L grade, and ambulatory physical activity, except for night pain. Results of full model ordinal logistic regression analyses for knee pain are provided in the Additional file 1: Table S2.

**Table 2:** Results of ordinal logistic regression analysis to characterize the association between depression and knee pain intensity (n = 95)^a^

Variables	Level of task-specific pain, no (%)			Proportional OR (95% CI)	*p*-value
	No	Mild	Moderate/Severe		
JKOM “pain and stiffness” score				**3.01 (1.37–6.62)**	**0.006**
*Task-specific knee pain*					
Do you feel stiffness in your knees when you wake up in the morning?					
*With* depression	15 (34.9)	13 (30.2)	15 (34.9)	**2.32 (1.02–5.31)**	**0.045**
*Without* depression	27 (51.9)	16 (30.8)	9 (17.3)		
Do you feel pain in your knees when you wake up in the morning?					
*With* depression	12 (27.9)	16 (37.2)	15 (34.9)	**2.42 (1.07–5.48)**	**0.033**
*Without* depression	24 (46.2)	19 (36.5)	9 (17.3)		
How often do you wake up in the night because of pain in your knees?					
*With* depression	21 (48.8)	11 (25.6)	11 (25.6)	1.92 (0.81–4.56)	0.141
*Without* depression	36 (69.2)	9 (17.3)	7 (13.5)		
Do you have pain in your knees when you walk on a flat surface?					
*With* depression	12 (27.9)	19 (44.2)	12 (27.9)	**2.87 (1.25–6.61)**	**0.013**
*Without* depression	27 (51.9)	18 (34.6)	7 (13.5)		
Do you have pain in your knees when ascending stairs?					
*With* depression	9 (20.9)	16 (37.2)	18 (41.9)	**3.73 (1.62–8.58)**	**0.002**
*Without* depression	26 (50.0)	17 (32.7)	9 (17.3)		
Do you have pain in your knees when descending stairs?					
*With* depression	9 (20.9)	16 (37.2)	18 (41.9)	**2.69 (1.19–6.10)**	**0.018**
*Without* depression	24 (46.2)	14 (26.9)	14 (26.9)		
Do you have pain in your knees when bending to floor or standing up?					
*With* depression	7 (16.3)	19 (44.2)	17 (39.5)	**2.41 (1.08–5.36)**	**0.031**
*Without* depression	18 (34.6)	22 (42.3)	12 (23.1)		
Do you have pain in your knees when standing?					
*With* depression	9 (20.9)	19 (44.2)	15 (34.9)	**3.99 (1.74–9.16)**	**0.001**
*Without* depression	30 (57.7)	13 (25.0)	9 (17.3)		

*JKOM* Japanese Knee Osteoarthritis Measure, *OR* Odds ratio; 95% CI: 95% confidence interval

^a^ Proportional OR (95% CI) for a greater quartile (JKOM pain and stiffness; 1–4; 1 [< 25th percentile] indicates mild pain and 4 [≥75 percentile] indicates severe pain) or greater task-specific knee pain (1: no pain, 2: mild pain, 3: moderate/severe pain) was calculated (continuous) to indicate predictive ability of the presence of depression while simultaneously including (one-step model) age (continuous), sex, body mass index (continuous), index knee radiographic tibiofemoral joint Kellgren/Lawrence grade (continuous), and objectively measured physical activity (continuous) in the ordinal regression model

See Additional file 1: Table S1 for details of quartiles in JKOM “pain and stiffness”

Bold represents statistically significant result

Ordinal logistic regression analysis further revealed (Table 3) that depression was significantly associated with a higher odds ratio of a greater quartile (i.e., severe disability) in the JKOM “activities of daily living” (proportional OR: 2.64; 95% CI: 1.18, 5.90; *p* = 0.018), gait velocity (proportional OR: 3.13; 95% CI: 1.37, 7.16; *p* = 0.007), and TUG (proportional OR: 3.12; 95% CI: 1.36, 7.16; *p* = 0.007), after being adjusted for age, sex, BMI, K/L grade, presence of bilateral knee pain, and ambulatory physical activity (Table 3). There was no significant association between depression and quartile of 5CS (proportional OR: 1.61; 95% CI: 0.75, 3.49; *p* = 0.223). Results of full model ordinal logistic regression analyses for physical function were provided in the Additional file 1: Table S3.

**Table 3:** Results of ordinal logistic regression analysis to characterize the association between depression and quartile of self-reported and performance-based physical function (n = 95) ^a^

Variables	Proportional OR (95% CI)	*p*-value
*Self-reported physical function*		
JKOM “activities of daily living”, points	**2.64 (1.18–5.90)**	**0.018**
*Performance-based physical function*		
Gait velocity, meters/second	**3.13 (1.37–7.16)**	**0.007**
Timed up and go, seconds	**3.12 (1.36–7.16)**	**0.007**
Five repetition chair stand, seconds	1.61 (0.75–3.49)	0.223

*JKOM* Japanese Knee Osteoarthritis Measure, *OR* Odds ratio; 95% CI: 95% confidence interval

^a^ Proportional OR (95% CI) for a greater quartile (1–4; 1 [< 25th percentile] indicates better function and 4 [≥75 percentile] indicates worse function) was calculated (continuous) to indicate predictive ability of the presence of depression while simultaneously including (one-step model) age (continuous), sex, body mass index (continuous), index knee radiographic tibiofemoral joint Kellgren/Lawrence grade (continuous), objectively measured physical activity (continuous), and presence of bilateral knee pain (0: absence, 1: presence) in the ordinal regression model

See Additional file 1: Table S1 for details of quartiles in JKOM “activities of daily living” and each performance-based physical function

Bold represents statistically significant result

## Discussion

The current study revealed that depression was significantly associated with worse knee pain and almost all worse task-specific knee pain during daily living except for night pain. Notably, contrary to our second hypothesis, depression was significantly associated with slower gait velocity and longer TUG time, even after considering covariates, such as bilateral knee pain and objectively measured ambulatory physical activity. Therefore, depression may be associated with increased knee pain intensity in a non-task-specific manner and in physical function.

Depressive symptoms are known to be factors associated with both knee pain and physical function, particularly self-reported physical function [6, 7], which we also observed. The differences in knee pain and self-reported physical function between patients with and without depression is approximately 10% of JKOM pain and functional subcategories. There correspond to clinically important meaningful differences, as defined by the Outcome Measures in Rheumatology Clinical Trials and OARSI [44], thereby indicating an important role of depression as a factor associated with knee pain and self-reported physical function. Importantly, depression has been suggested to be a more meaningful factor associated with knee pain and disability than radiographic evidence of degenerative joint changes [45–47]. Therefore, considering depressive symptoms may help resolve the discordance between radiographic findings and knee pain and disability [48]. We also found that there is no significant difference in K/L grade between patients with and without depression, which indicates minimal impact of radiographic severity on the relationship between depression and knee pain.

A significant finding of the present study is that depression was significantly associated with higher knee pain in non-task-specific manner. The association between depression and non-specific knee pain is counter to the theory that depression-related knee pain is mainly attributed to a nociceptive mechanism. Knee pain during weight bearing activities has been considered to be a nociceptive phenomenon (i.e., more supportable pressure being loaded in the knee joint during a weight bearing activity causes knee pain). Cumulative data suggest that, in addition to a nociceptive mechanism, central sensitization may contribute to knee pain in patients with knee OA [49, 50]. Psychological factors, including depression, are known contributors to OA pain [6, 7] and may further contribute to the maintenance of central sensitization, thereby lowering the pain threshold and increasing the likelihood of experiencing resting pain.

Interestingly, individuals with depressive symptoms had a non-significant association with night pain. Greater knee pain at night causes poorer sleep quality at night and feeling less refreshed after sleep [51], which may exacerbate depressive symptoms. Our findings challenge the theory that individuals with greater pain at night had disturbed sleep quality and subsequent exacerbation of depressive symptoms. However, this result should be interpreted with caution. This exploratory study did not perform pre-study sample size calculations, although we initially checked the maximum number of independent variables included in the ordinal logistic regression model. Therefore, a lack of statistical power due to a small number of included patients may explain this absence. Indeed, post-hoc power calculation detected by the Power and Sample Size Program, PS (version 3.1.2) [52] revealed that we have only 69.0% power to detect a standardized mean difference of at least 0.51, at the 5% alpha level. The lower 95% CI of proportional OR for the presence of night pain is close to 1, suggesting that further studies with larger sample sizes would be warranted to confirm the relationship between depressive symptom and night pain.

There is conflicting evidence regarding the association of depression with performance-based physical function [11–14]. Our results reinforce the observed negative impact of depressive symptoms on performance-based physical function. Notably, approximately 50% of patients with depression had bilateral knee pain. This is a significantly higher percentage than in patients without depression. Creamer et al. showed that injection of intra-articular anaesthetic in one knee decreased knee pain perception in both knees [53], which indicates that the descending pain pathways may modulate the pain perception of contralateral knee, rather than a systemic effect of the anaesthetic due to rapid dilution. Depression may lead to changes in neurologic pain pathways, which are attributable to a higher likelihood of bilateral knee pain. It is noteworthy that the significant association between depression and slower gait velocity and longer time of TUG were comparable before and after adjustment for covariates, including bilateral knee pain, in the logistic regression model (data not shown). This suggests a minimal role of bilateral knee pain on performance-based physical function in the present study. These results are contrary to previous studies that bilateral knee pain influence functional limitations [16, 17]. The cause of these discordances is unclear, however, relatively mild pain and functional status compared to previous studies may attribute to the results.

Interestingly, we found that ambulatory physical activity was not significantly different between patients with and without depression (Table 1). Our results are inconsistent with a well-known model (“avoidance model”) of activities [54] (i.e., psychological distress enhances the tendency to avoid daily activities, resulting in muscle weakness). Since most patients with knee OA are not physically active [55], and since engaging in ambulatory PA is critical to long-term independent living for patients with knee OA, more research is needed to understand the association between depression and physical activity.

### Study limitations

It is important to acknowledge that the cross-sectional nature of our study limits the ability to determine a causal relationship between depression and task-specific knee pain/physical function. Longitudinal studies in a large population show a bidirectional relationship between depression and slower gait speed [56], and slower gait speed is a predictor of chronicity [57] or worsening depression [58]. A prospective longitudinal study is warranted to determine the nature of the bidirectional relationship between depression and multiple physical functions. Second, only participants who responded to a distributed advertisement were included in the analyses, which may attribute to higher prevalence of depression (45.3%) than that in previous studies [4]. Furthermore, approximately 74% participants had mild radiographic OA in the present study; therefore, the participants may not be representative of a general population with knee OA and results should be interpreted with caution when translated to those with severe OA. Nevertheless, the relationships between depressive symptoms and worse knee pain/function were significant even after adjustment for covariates including K/L grade, indicating that these relationships were independent from radiographic OA severity. Third, quadriceps strength was not evaluated in this study as a covariate, despite having been demonstrated to be correlated with functional measures [59, 60]. Depression may result in avoidance of activities, thereby resulting in muscle weakness that may have a negative effect on physical function [54]. Finally, pain catastrophizing data was not evaluated in this study. Pain catastrophizing is the tendency to focus on and magnify pain sensations and to feel helpless in the face of pain [61]. Determining whether pain catastrophizing modulates the association between depression and physical function would be of interest.

## Conclusions

Depression was significantly associated with worse knee pain in non-task-specific manner. Furthermore, depression was significantly associated with slower gait velocity and a longer TUG time, even after controlling for covariates, such as bilateral knee pain and ambulatory physical activity. Our results reinforce the negative impact of depressive symptoms on knee pain and physical function in individuals with knee OA.

## Additional file

Additional file 1: **Table S1.** Quartile of each functional measure (greater quartile indicates worse knee pain or physical function), **Table S2.** Results of ordinal logistic regression analysis (including the results of covariates) to characterize the association between depression and knee pain intensity (*n* = 95)*. **Table S3.** Results of ordinal logistic regression analysis (including the results of covariates) to characterize the association between depression and quartile of self-reported and performance-based physical function (*n* = 95)*. (DOCX 61 kb)

## Abbreviations

5CS: Five repetition chair stand
BMI: Body mass index
GDS: Geriatric Depression Scale
JKOM: Japanese Knee Osteoarthritis Measure
K/L grade: Kellgren/Lawrence grade
OA: Osteoarthritis
OARSI: Osteoarthritis Research Society International
OR: Odds ratio
TUG: Timed up and go
