# Supplementary material for: Psychological health is associated with knee pain and physical function in patients with knee osteoarthritis: an exploratory cross-sectional study
Source: BMC Psychol. 2018 May 2;6:19. doi: 10.1186/s40359-018-0234-3 (PMC5930799; doi:10.1186/s40359-018-0234-3)
Supplement: Supplementary file 1 — Table S1. Quartile of each functional measure (greater quartile indicates worse knee pain or physical function), Table S2. Results of ordinal logistic regression analysis (including the results of covariates) to characterize the association between depression and knee pain intensity (n = 95)*. Table S3. Results of ordinal logistic regression analysis (including the results of covariates) to characterize the association between depression and quartile of self-reported and performance-based physical function (n = 95)*. (DOCX 61 kb) [file 40359_2018_234_MOESM1_ESM.docx]

Table S1. Quartile of each functional measure (greater quartile indicates worse knee pain or physical function)

| Variables | Quartile | | | |
| --- | --- | --- | --- | --- |
|  | < 25th percentile | 25–50th percentile | 50–75th percentile | ≥75 percentile |
| *Pain* |  |  |  |  |
| JKOM "pain and stiffness", points | 0–1 (n = 24) | 2–5 (n = 19) | 6–11 (n = 27) | 12–29 (n = 25) |
| *Self-reported physical function* |  |  |  |  |
| JKOM "activities of daily living", points | 0–1 (n = 20) | 2–5 (n = 23) | 6–11 (n = 28) | 12–30 (n = 24) |
| *Performance-based physical function* |  |  |  |  |
| Gait velocity, meters/second | 1.57–1.23 (n = 27) | 1.22–1.09 (n = 21) | 1.08–0.95 (n = 23) | 0.94–0.39 (n = 24) |
| Timed up and go, seconds | 5.03–6.65 (n = 23) | 6.66–7.68 (n = 23) | 7.69–9.31 (n = 25) | 9.32–21.8 (n = 24) |
| Five repetition chair stand, seconds | 3.66–7.56 (n = 23) | 7.57–8.66 (n = 24) | 8.67–10.1 (n = 24) | 10.2–14.5  (n = 24) |

JKOM: Japanese Knee Osteoarthritis Measure.

Table S2 Results of ordinal logistic regression analysis (including the results of covariates) to characterize the association between depression and knee pain intensity (n = 95)*

| Variables | Proportional OR (95% CI) | *p*-value |
| --- | --- | --- |
| JKOM "pain and stiffness" score |  |  |
| Depression (0: no, 1: yes) | **3.01 (1.37–6.62)** | **0.006** |
| Age, per years | 0.99 (0.94–1.04) | 0.608 |
| Female sex (0: no, 1: yes) | 0.81 (0.36–1.84) | 0.618 |
| BMI, per unit | 1.11 (0.99–1.24) | 0.079 |
| Index knee K/L grade, per grade | **2.14 (1.36–3.35)** | **<0.001** |
| Ambulatory physical activity, per steps/day | 1.06 (0.90–1.24) | 0.488 |
|  |  |  |
| *Task-specific knee pain* |  |  |
| Do you feel stiffness in your knees when you wake up in the morning? |  |  |
| Depression (0: no, 1: yes) | **2.32 (1.02–5.31)** | **0.045** |
| Age, per years | 1.02 (0.96–1.07) | 0.558 |
| Female sex (0: no, 1: yes) | 0.42 (0.17–1.02) | 0.055 |
| BMI, per unit | 1.10 (0.97–1.24) | 0.134 |
| Index knee K/L grade, per grade | **2.43 (1.51–3.92)** | **<0.001** |
| Ambulatory physical activity, per 1000 steps/day | 1.06 (0.89–1.26) | 0.528 |
|  |  |  |
| Do you feel pain in your knees when you wake up in the morning? |  |  |
| Depression (0: no, 1: yes) | **2.42 (1.07–5.48)** | **0.033** |
| Age, per years | 1.03 (0.97–1.09) | 0.307 |
| Female sex (0: no, 1: yes) | 0.68 (0.29–1.62) | 0.383 |
| BMI, per unit | 1.08 (0.96–1.22) | 0.207 |
| Index knee K/L grade, per grade | **2.49 (1.54–4.03)** | **<0.001** |
| Ambulatory physical activity, per 1000 steps/day | 1.09 (0.92–1.29) | 0.315 |
|  |  |  |
| How often do you wake up in the night because of pain in your knees? |  |  |
| Depression (0: no, 1: yes) | 1.92 (0.81–4.56) | 0.141 |
| Age, per years | 1.04 (0.98–1.10) | 0.202 |
| Female sex (0: no, 1: yes) | 0.97 (0.39–2.42) | 0.945 |
| BMI, per unit | 1.13 (0.99–1.28) | 0.057 |
| Index knee K/L grade, per grade | **1.80 (1.13–2.87)** | **0.013** |
| Ambulatory physical activity, per 1000 steps/day | 1.04 (0.87–1.24) | 0.681 |
|  |  |  |
| Do you have pain in your knees when you walk on a flat surface? |  |  |
| Depression (0: no, 1: yes) | **2.87 (1.25–6.61)** | **0.013** |
| Age, per years | 0.98 (0.93–1.03) | 0.444 |
| Female sex (0: no, 1: yes) | 1.13 (0.47–2.72) | 0.779 |
| BMI, per unit | 1.06 (0.94–1.19) | 0.368 |
| Index knee K/L grade, per grade | **2.92 (1.78–4.80)** | **<0.001** |
| Ambulatory physical activity, per 1000 steps/day | 1.03 (0.87–1.22) | 0.702 |
|  |  |  |
| Do you have pain in your knees when ascending stairs? |  |  |
| Depression (0: no, 1: yes) | **3.73 (1.62–8.58)** | **0.002** |
| Age, per years | 0.98 (0.93–1.04) | 0.444 |
| Female sex (0: no, 1: yes) | 1.04 (0.44–2.49) | 0.923 |
| BMI, per unit | 1.09 (0.96–1.22) | 0.171 |
| Index knee K/L grade, per grade | **2.41 (1.48–3.91)** | **<0.001** |
| Ambulatory physical activity, per 1000 steps/day | 1.08 (0.91–1.27) | 0.394 |
|  |  |  |
| Do you have pain in your knees when descending stairs? |  |  |
| Depression (0: no, 1: yes) | **2.69 (1.19–6.10)** | **0.018** |
| Age, per years | 0.99 (0.94–1.04) | 0.652 |
| Female sex (0: no, 1: yes) | 0.77 (0.33–1.83) | 0.555 |
| BMI, per unit | 1.11 (0.99–1.25) | 0.082 |
| Index knee K/L grade, per grade | **2.42 (1.49–3.93)** | **<0.001** |
| Ambulatory physical activity, per 1000 steps/day | 1.02 (0.87–1.21) | 0.793 |
|  |  |  |
| Do you have pain in your knees when bending to floor or standing up? |  |  |
| Depression (0: no, 1: yes) | **2.41 (1.08–5.36)** | **0.031** |
| Age, per years | 1.01 (0.95–1.06) | 0.851 |
| Female sex (0: no, 1: yes) | 0.91 (0.39–2.09) | 0.821 |
| BMI, per unit | 1.08 (0.97–1.22) | 0.175 |
| Index knee K/L grade, per grade | **1.70 (1.09–2.66)** | **0.019** |
| Ambulatory physical activity, per 1000 steps/day | 0.95 (0.81–1.11) | 0.522 |
|  |  |  |
| Do you have pain in your knees when standing? |  |  |
| Depression (0: no, 1: yes) | **3.99 (1.74–9.16)** | **0.001** |
| Age, per years | 1.01 (0.95–1.06) | 0.824 |
| Female sex (0: no, 1: yes) | 0.64 (0.27–1.54) | 0.322 |
| BMI, per unit | 1.03 (0.91–1.16) | 0.657 |
| Index knee K/L grade, per grade | **2.14 (1.34–3.41)** | **0.019** |
| Ambulatory physical activity, per 1000 steps/day | 1.10 (0.93–1.30) | 0.522 |

BMI: Body mass index; JKOM: Japanese Knee Osteoarthritis Measure; K/L grade: Kellgren/Lawrence grade; OR: Odds ratio; 95% CI: 95% confidence interval.

* Proportional OR (95% CI) for a greater quartile (JKOM pain and stiffness; 1–4; 1 [< 25th percentile] indicates mild pain and 4 [≥75 percentile] indicates severe pain) or greater task-specific knee pain (1: no pain, 2: mild pain, 3: moderate/severe pain) was calculated (continuous) to indicate predictive ability of the presence of depression while simultaneously including (one-step model) age (continuous), sex, body mass index (continuous), index knee radiographic tibiofemoral joint Kellgren/Lawrence grade (continuous), and objectively measured physical activity (continuous) in the ordinal regression model.

See Supplementary Table 1 for details of quartiles in JKOM “pain and stiffness”.

Bold represents statistically significant result.

Table S3. Results of ordinal logistic regression analysis (including the results of covariates) to characterize the association between depression and quartile of self-reported and performance-based physical function (n = 95)*

| Variables | Proportional OR (95% CI) | *p*-value |
| --- | --- | --- |
| JKOM "activities of daily living", points |  |  |
| Depression (0: no, 1: yes) | **2.64 (1.18–5.90)** | **0.018** |
| Age, per years | **1.07 (1.02–1.13)** | **0.012** |
| Female sex (0: no, 1: yes) | 1.47 (0.64–3.38) | 0.360 |
| BMI, per unit | 1.10 (0.98–1.24) | 0.095 |
| Index knee K/L grade, per grade | **1.78 (1.14–2.77)** | **0.011** |
| Ambulatory physical activity, per steps/day | 1.02 (0.88–1.20) | 0.759 |
| Presence of bilateral knee pain (0: absence, 1: presence) | **2.91 (1.28–6.64)** | **0.011** |
|  |  |  |
| Gait velocity, meters/second |  |  |
| Depression (0: no, 1: yes) | **3.13 (1.37–7.16)** | **0.007** |
| Age, per years | **1.15 (1.08–1.22)** | **<0.001** |
| Female sex (0: no, 1: yes) | 0.54 (0.23–1.26) | 0.154 |
| BMI, per unit | 1.12 (0.99–1.26) | 0.059 |
| Index knee K/L grade, per grade | 1.44 (0.93–2.24) | 0.102 |
| Ambulatory physical activity, per steps/day | 0.98 (0.84–1.16) | 0.848 |
| Presence of bilateral knee pain (0: absence, 1: presence) | 0.90 (0.40–2.04) | 0.802 |
|  |  |  |
| Timed up and go, seconds |  |  |
| Depression (0: no, 1: yes) | **3.12 (1.36–7.16)** | **0.007** |
| Age, per years | **1.18 (1.11–1.26)** | **<0.001** |
| Female sex (0: no, 1: yes) | 2.27 (0.96–5.36) | 0.062 |
| BMI, per unit | **1.14 (1.01–1.28)** | **0.031** |
| Index knee K/L grade, per grade | 1.30 (0.84–2.01) | 0.241 |
| Ambulatory physical activity, per steps/day | 0.93 (0.80–1.10) | 0.406 |
| Presence of bilateral knee pain (0: absence, 1: presence) | 0.64 (0.28–1.47) | 0.296 |
|  |  |  |
| Five repetition chair stand, seconds |  |  |
| Depression (0: no, 1: yes) | 1.61 (0.75–3.49) | 0.223 |
| Age, per years | **1.07 (1.01–1.12)** | **0.015** |
| Female sex (0: no, 1: yes) | 1.09 (0.49–2.42) | 0.837 |
| BMI, per unit | 1.08 (0.97–1.21) | 0.172 |
| Index knee K/L grade, per grade | 0.89 (0.59–1.34) | 0.577 |
| Ambulatory physical activity, per steps/day | 1.06 (0.91–1.23) | 0.453 |
| Presence of bilateral knee pain (0: absence, 1: presence) | 0.98 (0.45–2.13) | 0.959 |

BMI: Body mass index; JKOM: Japanese Knee Osteoarthritis Measure; K/L grade: Kellgren/Lawrence grade; OR: Odds ratio; 95% CI: 95% confidence interval.

* Proportional OR (95% CI) for a greater quartile (1–4; 1 [< 25th percentile] indicates better function and 4 [≥75 percentile] indicates worse function) was calculated (continuous) to indicate predictive ability of the presence of depression while simultaneously including (one-step model) age (continuous), sex, body mass index (continuous), index knee radiographic tibiofemoral joint Kellgren/Lawrence grade (continuous), objectively measured physical activity (continuous), and presence of bilateral knee pain (0: absence, 1: presence) in the ordinal regression model.

See Supplementary Table 1 for details of quartiles in JKOM “activities of daily living” and each performance-based physical function.

Bold represents statistically significant result.
